# Role of Oxidative Stress in Pathophysiology of Nonalcoholic Fatty Liver Disease

**DOI:** 10.1155/2018/9547613

**Published:** 2018-06-11

**Authors:** Mario Masarone, Valerio Rosato, Marcello Dallio, Antonietta Gerarda Gravina, Andrea Aglitti, Carmelina Loguercio, Alessandro Federico, Marcello Persico

**Affiliations:** ^1^Internal Medicine and Hepatology Division, Department of Medicine, University of Medicine of Salerno, Salerno, Italy; ^2^Hepatogastroenterology Division, University of Campania “Luigi Vanvitelli”, Naples, Italy

## Abstract

Liver steatosis without alcohol consumption, namely, nonalcoholic fatty liver disease (NAFLD), is a common hepatic condition that encompasses a wide spectrum of presentations, ranging from simple accumulation of triglycerides in the hepatocytes without any liver damage to inflammation, necrosis, ballooning, and fibrosis (namely, nonalcoholic steatohepatitis) up to severe liver disease and eventually cirrhosis and/or hepatocellular carcinoma. The pathophysiology of fatty liver and its progression is influenced by multiple factors (environmental and genetics), in a “multiple parallel-hit model,” in which oxidative stress plays a very likely primary role as the starting point of the hepatic and extrahepatic damage. The aim of this review is to give a comprehensive insight on the present researches and findings on the role of oxidative stress mechanisms in the pathogenesis and pathophysiology of NAFLD. With this aim, we evaluated the available data in basic science and clinical studies in this field, reviewing the most recent works published on this topic.

## 1. Introduction

The presence of a significant (>5% of hepatocytes) fat accumulation in the liver, in the absence of an “unsafe” quantity of alcohol consumption and any other cause of liver diseases, is a potentially pathological condition that is defined as nonalcoholic fatty liver disease (NAFLD) [1]. In the last two decades, it has become the most “emerging” liver disease worldwide, since we are moving towards a gradual reduction of viral hepatitis and a progressive increase of obesity, following the spread of a “western lifestyle” [2, 3]. A wide pathological spectrum of liver injury is associated with NAFLD, ranging from indolent steatosis, usually characterized by an asymptomatic benign clinical course, to nonalcoholic steatohepatitis (NASH), which can lead to fibrosis with an evolutionary course in cirrhosis and hepatocellular carcinoma (HCC) [4–6]. Alarmingly, in patients with NASH, HCC can also develop itself without first progressing to cirrhosis [7].

The prevalence of NAFLD is estimated to be as high as 17–33% in the general population, while it reaches 75% in obese individuals and even more in patients with type 2 diabetes mellitus (T2DM) [3, 8]. The concomitant presence of T2DM increases the risk of progression of liver damage and constitutes a significant risk for cardiovascular diseases [9].

Although obesity, particularly central (abdominal) obesity, is a well-recognized risk factor for it, NAFLD has been also reported in lean individuals (body mass index < 30 kg/m^2^) [10]. Furthermore, the prevalence of NAFLD differs depending on the gender, ethnicity, and race as a proof of probable involvement of genetic and epigenetic factors in the pathogenesis of the disease [3, 11, 12].

Insulin resistance (IR) is the major pathophysiological factor implicated in NAFLD, as well as metabolic syndrome (MS), a cluster of cardiovascular risk factors comprising visceral obesity, blood hypertension, glucose intolerance, and dyslipidemia [13]. In this way, NAFLD has been considered as the liver expression of MS, not only burdened with high cardiovascular risk but also responsible of a progressive metabolic, cardiovascular, and/or kidney disease, even without an overt MS [14].

IR is thought to play a pivotal role both to the initiation of the disease and the pathogenic switch of fatty liver to advanced forms of NAFLD, even if the mechanisms underlying this process are still partially unknown [15]. The pathophysiologic mechanism was firstly hypothesized by Day and James who proposed the “two-hit model,” in which the simple steatosis (the first “hit”) in addition to other factors (that were primarily linked to the increase in oxidative stress) were needed for the development of NASH (the second “hit”) [16]. This first hypothesis has been subsequently revised in a “multiple parallel-hit” model in which, in the presence of a significant accumulation of fat in hepatocytes and systemic and hepatic insulin resistance, multiple simultaneous alterations lead to an imbalance between the antilipotoxic protection system of the liver (mitochondrial betaoxidation) and the free radical production in gut and adipose tissue, resulting in endoplasmic stress, oxidative stress, and hepatocyte apoptosis [17].

In this paper, we will review the main evidences on the strict pathophysiologic linkage between oxidative stress mechanisms and the presence NAFLD and its progression, particularly focusing on the most reported pathophysiological mechanisms: mitochondrial dysfunction, endoplasmic reticulum (ER) stress, iron metabolism derangements, gut-liver axis, insulin resistance, and endothelial dysfunction.

## 2. Mitochondrial Dysfunction

Since the first studies on NAFLD, many evidences pointed out that it was primarily characterized by the presence of mitochondrial dysfunction [18]. The homeostasis of fat and energy in hepatic cells is regulated by mitochondrial activities, including betaoxidation of free fatty acids (FFAs), electron transfer and production of ATP, and reactive oxygen species (ROS) [19]. Mitochondrial abnormalities alter the balance between prooxidant and antioxidant mechanisms, leading to an increase of nonmetabolized fatty acids in the cytosol as a result of the blockade of fatty acid betaoxidation and the consequent induction of ROS production [20]. The alteration of mitochondrial functions is evident with electron microscopy analysis by some ultrastructural changes such as megamitochondria, loss of cristae, and paracrystalline inclusion bodies in the matrix [21].

An intriguing hypothesis is that a “primary” mitochondrial dysfunction may be the initiator of accumulation of fatty acids in the hepatocytes during insulin-resistance-associated NAFLD particularly if a fat-rich diet provides an increased supply to the liver [22]. The “primary” mitochondrial dysfunction should be due to multiple mechanisms, ranging from mitochondrial DNA damage to sirtuin imbalance. Damage of mitochondrial DNA (mtDNA) and nucleic genes encoding mitochondrial proteins may involve a progressive increase of oxidative stress levels, as in Alpers-Huttenlocher disease, an autosomal-recessive hepatocerebral syndrome, due to a polymerase gamma mutation and mtDNA depletion, that causes the development of liver steatosis until end-stage liver failure [23]. Moreover, in a murine model was shown that the mutation of the gene encoding for mitochondrial isobutyryl-CoA-dehydrogenase is associated with a hepatic steatosis [24]. Sirtuins are a group of NAD(+)-dependent deacetylase, involved in oxidative damage of both alcoholic and nonalcoholic fatty liver diseases [25]. SIRT1, the most studied of these enzymes, has an indirect regulatory effect on oxidative stress, activating forkhead proteins and PGC-1, transcription factors involved in transcription of antioxidant enzyme genes and in ROS-detoxifying capacity [26]. SIRT3 can increase betaoxidation of FFAs by activation of long-chain acyl-CoA dehydrogenases, and its activity was found decreased in animal models with fatty liver [27]. In this way, a primary decrease of SIRT3, associated with a fat-rich diet, can initiate a pathological process. Sirtuins 1 and 3 using NAD+ as the cosubstrate elicits various metabolic improvements in many tissues (lung, spleen, brain, and small intestine) [28]. In this way, NAD+ depletion may contribute to mitochondrial dysfunction obstructing the adaptive response mediated by sirtuins to high FFA hepatic levels [29].

Besides the hypothesis of a primary mitochondrial dysfunction, it is noted that, during the early phase of fatty infiltration in the hepatocytes, several adaptive metabolic mechanisms are mediated by mitochondrial activity, with the aim of partitioning the lipotoxic FFAs into stable intracellular triglyceride stores, in order to prevent oxidative stress and ROS production [30]. This hypothesis was confirmed in an experimental model of NAFLD in which the mice were subjected to a methionine- and choline-deficient diet, after inhibiting the expression of diacylglycerol O-acyltransferase 2 (DGAT2), the enzyme catalyzing the final step of conversion of FFAs into triglycerides. In this study, despite a reduction of hepatic steatosis, it showed an increase of lipid oxidant stress markers and a worsening of lobular necroinflammation and fibrosis compared to controls, suggesting that steatosis may be a protective mechanism to prevent the progression of liver damage in NAFLD [31].

**Figure 1:**
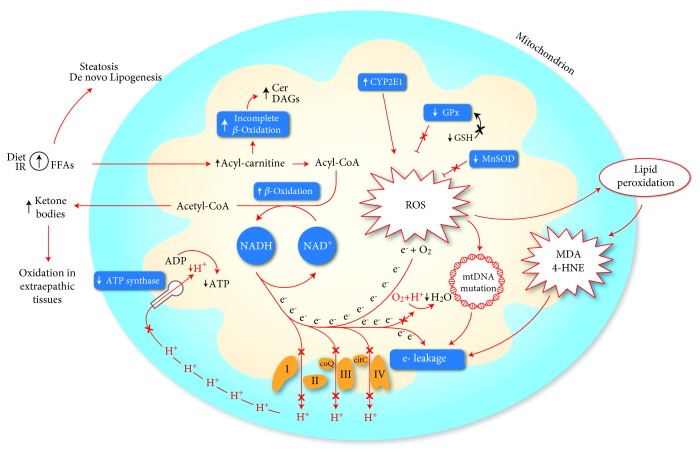
Mechanisms of mitochondrial dysfunction involved in the production of oxidative stress. An increase of mitochondrial betaoxidation activity, due to a lipid overload, may induce an impairment of electron transport chain, resulting in an “electron leakage.” The reaction between oxygen and protons catalyzed by cytochrome C oxidase (VI complex) is impaired, and electrons may interact directly with oxygen forming ROS. Furthermore, the generation of mitochondrial membrane potential is reduced following the reduction of proton extrusion from the matrix, weakening the activity of ATP synthase. ROS production may exacerbate the mitochondrial dysfunction due to electron leakage following the generation of mtDNA mutation and highly reactive aldehydes (MDA, 4-HNE) produced through lipid peroxidation. Mitochondrial CYP2E1 is a direct source of ROS. A reduction of antioxidant mechanism as GPx and MnSOD was also observed in the NASH model. At last, the incomplete suboptimal oxidation of acyl-carnitine leads to accumulation of lipotoxic intermediates (Cer, DAGs), which can act as an inflammatory intermediate altering the insulin signaling. 4-HNE: 4-hydroxy-2-nonenal; Cer: ceramides; CYP2E1: cytochrome P450 2E1; DAGs: diacylglycerols; FFAs: free fatty acids; GPx: glutathione peroxidase; GSH: glutathione; MDA: malondialdehyde; MnSOD: manganese superoxide dismutase; ROS: reactive oxygen species.

An overload of FFAs into mitochondria, subsequent to an increased intake or an insulin-resistance condition, even if it reduces the FAA cytosolic concentration, may lead to an increase in the permeability of the inner mitochondrial membrane. This occurrence leads to the dissipation of the membrane potential and the loss of ATP synthesis capacity, resulting in a mitochondrial function impairment and an enhanced ROS generation. The increase of fatty acid oxidation, inducing an increased electron flux in the electron transport chain (ETC), may generate an “electron leakage” (due to reduction of the activity of ETC complexes), thus ensuring a direct reaction between electrons and oxygen, leading to the formation of ROS, rather than the normal reaction mediated by cytochrome C oxidase that combines oxygen and protons in order to form water [19]. The incomplete or suboptimal betaoxidation leads to accumulation of long-chain acylcarnitines, ceramides, and diacylglycerols, lipotoxic intermediates that may promote inflammation and alter the insulin signaling [32]. It was demonstrated that these prooxidant mechanisms act as indirect sources of ROS. The loss of cytochrome C by mitochondria produces threefold more hydrogen peroxide (H_2_O_2_) than nondepleted mitochondria [33]; an impaired function of complex I-linked respiration, in a rodent model fed with choline-deficient diet, showed an abnormal ROS production [34]. Alpha-ketoglutarate dehydrogenase, growth factor adapter p66-Shc, monoamine oxidase, and pyruvate dehydrogenase may be other indirect sources of ROS, contributing to increased oxidative stress (Figure 1). Moreover, mitochondrial cytochrome P450 2E1 (CYP2E1), which is a potential direct source of ROS, has been demonstrated to have an increased activity in an animal model of NASH and also in NASH patients [35, 36]. Also CYP2E1, which is responsible for long-chain fatty acid metabolism, produces oxidative radicals and could act as a part of the “second hit” of the pathophysiological mechanism of NAFLD [37]. Furthermore, some CYP2E1 polymorphisms, particularly the c2 allele, has been shown to be associated with the development of NASH in obese, nondiabetic subjects, paving the way for a possible explanation about why not all the subjects with NAFLD go on to develop NASH [38].

In addition to prooxidant mechanism, in an experimental model of NASH, a decreased activity of several detoxifying enzymes was observed. Glutathione peroxidase (GPx) activity is reduced probably in consequence of GSH depletion and impaired transport of cytosolic GSH into the mitochondrial matrix [39]. The polymorphism C47T of the SOD2 gene, encoding for manganese superoxide dismutase, is associated with a reduction of activity of this enzyme resulting in an increased ROS production and a high susceptibility to developing NASH and advanced fibrosis in NAFLD [40].

The initial mitochondrial dysfunction can be further exacerbated by the production of mtDNA mutation by ROS and highly reactive aldehydes, such as malondialdehyde (MDA) and 4-hydroxy-2-nonenal (4-HNE), through lipid peroxidation following the interaction between ROS and polyunsaturated FAs. Cytochrome C oxidase may be directly inhibited by MDA while 4-HNE may contribute to “electron leakage” uncoupling the complex 2 of the electron transport chain. The ETC oxidative capacity may be also decreased by derivative damage by interaction between mitochondrial membranes and both MDA and 4-HNE [41].

### 2.1. Other Mechanisms of Oxidation

#### 2.1.1. ER Stress

Some evidences in the literature indicated that saturated fatty acids may have a more damaging effect on hepatocytes and liver function compared to unsaturated fatty acids, probably because of the ability of the latter to be esterified into triglycerides, which prevents fatty acid toxicity [42–46]. Saturated fatty acids determine several mechanisms of liver injury including the disruption of endoplasmic reticulum (ER) homeostasis, namely, ER stress, from which various proinflammatory pathways can be activated, very often culminating in cell death. ER is involved in folding and assembling proteins to attain their final appropriate conformation, but under stress conditions, such as lipid overload, the unfolded and misfolded proteins accumulate in the ER lumen, activating a specific signaling pathway called the unfolded protein response (UPR), in order to restore ER homeostasis [47]. UPR is regulated by three transmembrane stress transducers, protein kinase RNA-like ER kinase (PERK), activating transcription factor 6 (ATF6), and inositol-requiring signaling protein 1 (IRE1). First of all, the cumulative response to these signaling pathways blocks the initiation of translation, in order to reduce the load upon ER. Subsequently, it induces the expression of ATP-requiring chaperon protein in order to correct the misfolded protein, creating a further energetic demand on the potentially dysfunctional mitochondrial, and so on. Lastly, it activates the cellular death signaling [48]. Moreover, in response to an excessive ER activity mediated by cellular stressor, the initiation of inflammatory and apoptotic pathways is started through activation of c-Jun N-terminal kinase (JNK), implicated in the expression of hepatic insulin resistance [49]. In fact, it has been demonstrated that the suppression of the JNK1 can prevent the development of steatohepatitis in MCD diet-fed mice, explaining the importance of JNK signaling in the pathogenesis of this disease [50].

Furthermore, ER is a potent source of ROS and the formation of each disulfide bond during the oxidative folding of nascent proteins is associated with the production of a single ROS, and this process accounts for about the 25% of all cellular ROS generation [51]. ER-resident protein oxidoreductin 1 (ERO1) is essential in this process, and the expression of this flavoenzyme was found increased in an animal model of NAFLD, along with the other markers of inflammatory signaling activation, which we will discuss below [52].

A prolonged ER stress may lead to an increased UPR-mediated ROS generation by activation of proapoptotic protein CCAAT/enhancer-binding homologous protein (CHOP), which is regulated by PERK and ATF6 signaling pathways [53]. It was shown that CHOP deletion, in a mouse model of type 2 diabetes, improved glycemic control reducing levels of oxidative damage, suggesting that CHOP activation could enhance oxidative stress [48]. Recently, similar evidences have been produced also in mouse models of steatohepatitis [54]. In contrast of these results, a recent study attested that CHOP knockout mice, fed with a high-fat and cholesterol diet, had more severe histological features of NASH compared to wild types. Moreover, the treatment with liraglutide did not improve the insulin sensitivity in absence of CHOP, demonstrating also hepatoprotective mechanisms of this protein against ER stress [55].

Furthermore, the ER lumen is the main site of calcium storage, and calcium homeostasis plays a critical role in ER stress. Saturated fatty acids may induce a disruption of ER calcium store, which can act on mitochondrial membranes blocking ETC through the formation of permeability transition pores for cytochrome C, resulting in an increased ROS production and apoptosis induction [56, 57]. The calcium homeostasis is regulated also by sarco/endoplasmatic reticulum Ca^2+^-ATPase (SERCA) that pumps Ca^2^ into ER from the cytoplasm. The expression of SERCA was shown to be reduced in animal models of obesity and diabetes, and its inhibition was associated with the activation of ER stress response, resulting in UPR-mediated apoptotic pathways [58]. Conversely, it has been proven, in a mouse model of insulin resistance and type 2 diabetes, that the activation of SERCA can mediate reduction of adipose tissue, improvement in glucose tolerance, and hepatosteatosis, thus suggesting a promising therapeutic target in metabolic dysfunctions [59]. More recently, in a cellular model of hepatic steatosis, the role of protein kinase C *δ* (PKC*δ*) was demonstrated, whose activation by FFAs participates in fatty degeneration of hepatocytes during NASH, inducing ER stress and leading to CHOP-induced cell apoptosis [60]. The silencing of PKC*δ* was associated with downregulation of CHOP expression and an enhancement of SERCA activity, alleviating ER stress and stabilizing calcium homeostasis [61].

**Figure 2:**
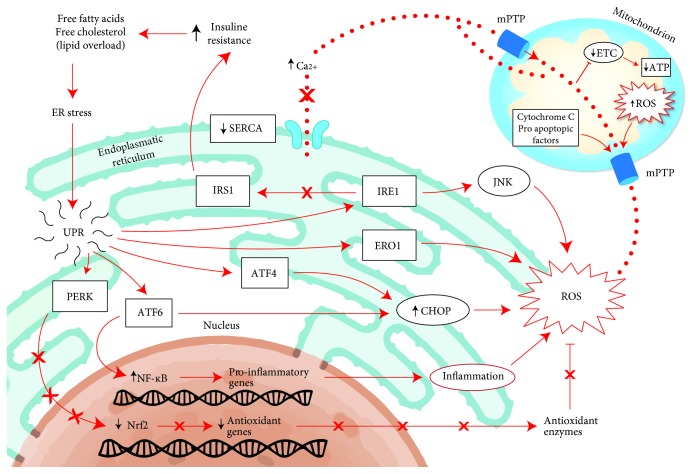
Pathway linked to ER stress. Schematic representations of hepatocyte in a fatty liver. A lipid overload, free fatty acids, and cholesterol may induce ER stress leading to “unfolded protein response” in order to reestablish the ER homeostasis. Its prolonged and repetitive activation should trigger a proapoptotic and inflammatory pathway resulting in an increase of oxidative stress. The activity of UPR is fundamentally mediated through three transmembrane stress transducers (PERK, IRE1, and ATF6), which regulate the expression of proinflammatory and antioxidant genes. In a prolonged ER stress condition, the ROS production is also increased by the overexpression of ERO1, an inducible ER oxidoreductase. Saturated fatty acids and ER stress can reduce the activity or SERCA determining a disruption of ER calcium store which can act on mitochondria blocking ETC and forming mPTP resulting in an uncontrolled transition of cytochrome C and other proapoptotic factors into the cytosol. ATF4: activating transcription factor 4; ATF6: activating transcription factor 6; CHOP: C/EBP homologous protein; ER: endoplasmatic reticulum; ERO1: ER oxidoreductin 1; ETC: electron transport chain; IRE1: inositol-requiring signaling protein 1; IRS1: insulin receptor substrate 1; mPTP: mitochondrial permeability transition pore; PERK: protein kinase RNA-like ER kinase; ROS: reactive oxygen species; SERCA: sarco/endoplasmic reticulum CA^2+^-ATPase; UPR: unfolded protein response.

Lastly, UPR was also involved in a decrease in antioxidant mechanism, as the reduction of glutathione levels. UPR mediates via PERK-signaling the inhibition of nuclear factor- (erythroid-derived 2-) like 2 (Nrf2). Nrf2 is a critical effector of cell survival, which activates transcription of antioxidant enzymes (glutathione S-transferase A2 and NADPH: quinone oxidoreductase 1), playing a critical role in elimination of ROS. In a high-fat-diet mouse model, it was demonstrated that the deletion of Nrf2 is associated with an oxidative stress increase by glutathione level decrease and catalase and superoxide-dismutase activity, resulting in rapid progression to NASH [62]. In Figure 2, these mechanisms are graphically shown.

## 3. Iron Metabolism Derangements

The first observations of an altered homeostasis of iron metabolism in the clinical profile of NASH date back to 1994, when it was reported that this condition is characterized by the presence of increased levels of ferritin and transferrin [63]. A mild hyperferritinaemia is found in at least the half of patients with NAFLD and often represents the only laboratory alteration that leads to the diagnosis of NAFLD. Recently, in a study conducted on 222 NAFLD patients followed for about 15 years, it was demonstrated that an elevated level of ferritin, in addition to being a strong predictor of advanced liver fibrosis, is associated with an increased long-term risk of death [64]. Iron metabolism is closely related to both oxidative stress and insulin resistance, 2 key points of NAFLD pathogenesis. The hemochromatosis (HFE) gene variants (particularly H63D and C828Y) were evaluated in cohorts of NASH patients in order to find a possible correlation, but it still remains unclear. In a large study on 786 patients, the subjects carrying these two genetic variations, despite showing a higher hepatocellular iron content, had less liver ballooning or NASH [65]. Conversely, in an older study on 51 NASH patients, a linear correlation between hepatic iron and severity of fibrosis in patients homozygous or heterozygous for C282Y was demonstrated. The study concluded that the presence of such mutation in NASH patients was associated with a higher content of hepatic iron and a significantly higher hepatic damage [66]. The hormone hepcidin inhibits the intestinal iron absorption and inactivates the cellular iron exporter ferroportin-1 [67]. In another study on 216 NAFLD patients, an association between the homozygosity for mutation p.Ala736Val in the gene of transmembrane protease serine 6 that led to a loss-of-function mutation of the enzymes matriptase-2 was found. This occurrence resulted in an increase of hepcidin expression, lower hepatic iron stores, and a decreased hepatocellular ballooning, typical features of oxidative stress in NAFLD [68].

The main prooxidizing mechanism generated by an unbound iron overload is characterized by the ability of the ferrous iron to catalyze, via Fenton reaction, the production of hydroxyl radical (OH^−^) from H_2_O_2_, whose cytosolic production derives predominantly by peroxisomal *β*-oxidation. Unlike the mitochondrial *β*-oxidation, the peroxisomal system is more active on long-chain and branched-chain FAs. Moreover, the peroxisomal acyl-CoA oxidase transfers electrons directly to oxygen and not to the respiratory chain (via electron-transferring flavoproteins, such as mitochondrial acyl-CoA dehydrogenase) generating H_2_O_2_, subsequently cleaved by catalase. In a mouse model study, it was demonstrated that iron overload can promote the activity of 15-lipoxygenase. This enzyme is able to induce the leakage of peroxisomal membrane, which is normally involved in the physiological turnover of these cytosolic organelles, but in a pathological iron overload as it occurs in NASH, it may be overactivated, resulting in an increase of H_2_O_2_ production [69]. This oxidative process may result in a lipid peroxidation that modifies the fatty acid profile of cellular membranes, leading to cell organelle damage and impairment of mitochondrial oxidative metabolism. Moreover, iron may directly catalyze lipid peroxidation, resulting in the production of malondialdehyde, which is involved in the fibrogenesis process by activation of hepatic stellate cells [67].

Furthermore, it has also been shown, in a rodent model, that chronic iron overload, obtained by diet enriched with carbonyl-iron, enhances the inducible nitric oxide synthase (iNOS) by extracellular signal-regulated kinase and NF-κB activation [70]. Iron overload can also reduce the antioxidant capacity decreasing GSH, therefore limiting GPx activity [70]. In the same way, a rat model of NASH was shown that parenteral administration of iron worsened steatosis and induced the development of fibrosis increasing apoptosis [71].

Conversely, it has been proven that iron depletion (rats fed an iron-deficient diet or treated with phlebotomy) may improve diabetic complications of oxidative stress by inhibitions of hepatic superoxide production and lipid peroxidation [72].

Nevertheless, the oxidizing effects of iron may also be indirectly mediated through the inhibition of the antioxidant mechanism, as heme oxygenase-1 (HO-1). HO-1 is the Kupffer cells' inducible isoform of heme oxygenase that possesses antioxidant/anti-inflammatory properties deriving from the elimination of heme and its reactive products, including biliverdin, bilirubin, and iron. HO-1 expression is increased in NAFLD and in mice under iron overload diet, as an adaptive response to oxidative damage [73, 74]. BACH-1 is a heme-binding factor able to repress the transcription of gene encoding for HO-1, and it was shown, in knockout BACH-1 mice fed with a methionine-choline-deficient diet, to have a hepatoprotective effect against steatohepatitis, probably due to failing to inhibit HO-1 [75]. In contrast, it was also demonstrated by Otogawa et al., in a rabbit model of steatohepatitis, a prooxidant mechanism of HO-1 trough-free iron generation by metabolism of heme, following the phagocytosis of erythrocyte in Kupffer cells, thereby enhancing hydroxyl radicals and lipid peroxides [76]. Interestingly, it has also been shown recently that an intermittent hypoxemia may have a limiting effect on hepatic steatosis. This occurs probably because the increase in hemoglobin levels promotes HO-1 expression with increased iron production, but this latter is rapidly sequestered by ferritin-1 and transferred into the bone marrow for erythropoiesis, limiting the production of hydroxyl radicals in the liver [77]. An inhibition of proinflammatory transcription factor NF-κB in mice subjected to intermittent hypoxia was also observed, suggesting that HO-1 contributes to the protection against liver injury, limiting inflammatory response.

**Figure 3:**
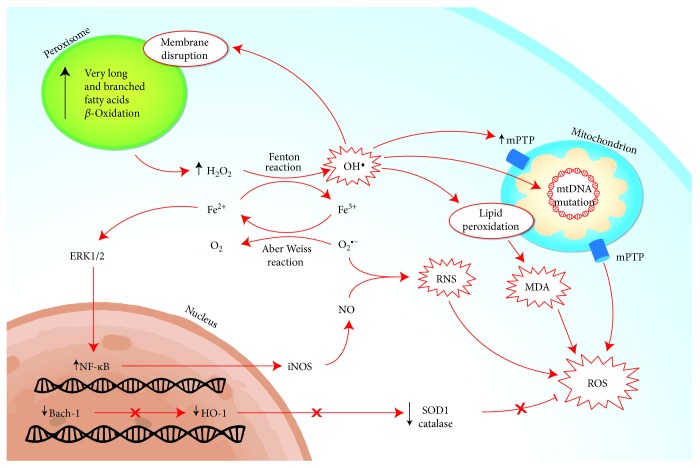
Mechanisms of oxidative stress induced by iron metabolism derangements. The main prooxidizing mechanism is characterized by the production of hydroxyl radical from H_2_O_2_ and Fe^2+^ via Fenton reaction. The main source of H_2_O_2_ is the peroxisomal betaoxidation of very long and branched fatty acids. The hydroxyl radicals may generate lipid peroxidation of organelle membranes, leading to impairment of mitochondrial metabolism through the production on mPTP and mtDNA mutation, but also increasing the proapopotitic activity with the production of MDA. Furthermore, the chronic iron overload may also enhance the production of iNOS via NF-κB activation, leading to an increase in nitric oxide and, consequently, to a reaction with the superoxide anion, RNS. At last, the antioxidant mechanism may be inhibited by iron overload, as the activation of BACH-1, a heme-binding factor able to repress the transcription of gene encoding for HO-1, that possesses antioxidant/anti-inflammatory properties. BACH-1: BTB and CNC homology 1; MDA: malondialdehyde; mPTP: mitochondrial permeability transition pore; HO-1: hemoxygenase 1; ROS: reactive oxygen species; RNS: reactive nitrogen species; SOD1: superoxide dismutase 1.

In response to the discrepant findings of Otogawa et al.'s study, it can be hypothesized that an increase of oxidative stress could also persist despite the overexpression of HO-1, if the amount of iron exceeds the capacity of iron-detoxification systems, including ferritin-1. In Figure 3, these mechanisms were graphically shown.

## 4. Gut-Liver Axis

The adult human intestine hosts a complex of enormous populations of bacteria, at least 100 trillion, defined as gut microbiota. The gut microbiota maintains a symbiotic relationship with the organism, providing genetic and metabolic attributes, in order to contribute to various functions, including digestion of otherwise inaccessible nutrients, vitamin synthesis, and resistance to colonization by pathogens [78]. The composition of the gut microbiota is variable and dynamic during human life, being influenced by several factors including diet, environmental hygiene, and antibiotic use and misuse [79]. Qualitative and quantitative modifications of “normal” gut microbiota, namely, dysbiosis, may be involved in development and progression of NAFLD, as well as other chronic metabolic diseases [80, 81]. Gut microbiota ferment some carbohydrates (as cellulose, xylans, and inulin, nondigestible by human enzymes) in order to produce short-chain fatty acids (SCFAs), lipid precursors but also effectors of metabolic pathways, with overall benefits on obesity [82]. In fact, SCFAs, inducing secretion of peptide YY, improve the extraction of calories from the gut, increasing the sense of satiety and thermogenesis, with a reduction of food intake and lipogenesis. Furthermore, SCFA ameliorates gut barrier function, reducing the permeability of bacterial toxins and metabolic endotoxemia that can induce inflammation and insulin resistance [83]. As a result of a condition of dysbiosis, SCFA production is reduced and FFA production from VLDL is increased. This is a consequence of the lack of lipoprotein lipase inhibition through the suppression of angiopoietin-related protein 4 by altered microbiota. Besides a higher lipid accumulation in the liver and the consequent steatosis, a dysbiosis, in human and rodent models, has been shown to be able to alter the integrity of the intestinal barrier, inducing bacteria and bacterial product translocation into the portal circulation, and the activation of proinflammatory pathways. That is the main mechanism associated with the progression of chronic liver disease [84, 85]. The activation of proinflammatory pathways is mediated by some hepatic receptors among which the Toll-like receptors (TLRs) are the best characterized. TLRs may mediate an inflammatory activation in response to recognition of some specific pathogen-associate molecular patterns (PAMPs), including lipopolysaccharide (bound by TLR4), bacterial flagellin (TLR5), double-stranded bacterial DNA (TLR9), or peptidoglycan (TLR2) [86]. The inflammatory signaling activated by TLRs take part in the activation of inflammasomes, a multimeric protein complex regulating the activation of caspase-1 that cleaves the precursor cytokines pro-IL1*β* and pro-IL18, whose activated cytokines are involved in inflammation and cell death [86]. Several evidences in literature demonstrated that the inflammasome activity drives NASH progression, and the inhibition of its activation may improve NAFLD pathology and fibrosis [87, 88]. Besides, some evidences from animal studies have shown that deficiency in inflammasome components, specifically NOD-like receptor proteins 3 (NPRL-3) and 6 (NPRL-6), results in reduced inflammatory response, inhibiting IL1*β* and IL18, and in alteration of gut homeostasis with loss of epithelial integrity, dysbiosis, and bacteraemia [89, 90]. In this way, the integrity of the gut microbiota composition seems to be closely correlated with the innate immune response mediated by the inflammasome and consequently with the inflammatory response from which may depend the progression of NAFLD. In an animal model study, it was demonstrated that a high-fat diet may increase the percentage of gram-negative bacteria expressing endotoxins, specifically Proteobacteria, within the gut, resulting in a higher bacterial translocation and in a higher degree of liver injury. Interestingly, though an increase of TLR expression and inflammasome component, as mRNA level of NPRL-3, was found upregulated in the liver of high-fat-diet mice in comparison to controls, an opposite trend was shown in the gut [91]. More recently, these data were reconfirmed in a high-fat- and high-carbohydrate-diet mice model, showing that an abundance of Verrucomicrobia and Proteobacteria was associated to a lack in NPRL-3 inflammasome and a higher hepatic expression of TLR4 and TLR9, resulting in a worse degree of liver injury that was restored after antibiotic treatment [92]. A study on obese and NASH human patients showed abundant representations of Bacteroides and Prevotella and a lower proportion of Firmicutes compared to healthy controls. Furthermore, an elevated blood-ethanol concentration was found exclusively in NASH patients, suggesting an abundance of ethanol-producing bacteria in their gut microbiome and a role of these bacteria in the pathogenesis of NASH [93]. On this topic, the following hypotheses have been drawn: that NAFLD is indeed an endogenous alcoholic fatty liver disease, considering that gut microbiota can produce daily a quantity of ethanol well above what is considered as safe for humans; that the hepatic first-pass metabolism of ethanol prevents the development of a detectable blood-alcohol concentration; and that, in patients with NASH, genes involved in the metabolism of endogenous ethanol are upregulated. This may particularly be for genes encoding for the hepatic alcohol dehydrogenase (ADH 4) that are strictly involved in liver exposure to high concentrations of ethanol [94].

The close correlation between intestinal dysbiosis and NAFLD has led to the hypothesis that a possible therapeutic option, alongside the use of probiotics and prebiotics, could be the faecal microbiota transplantation, which is already approved for the treatment of Clostridium difficile infection, giving promising results also in the treatment of metabolic syndrome and irritable bowel disease [95]. In favor of therapeutic applicability of faecal microbiota transplantation, some promising evidences emerged from preanalytical studies on the effects on insulin sensitivity and systemic inflammation, but the clinical trials are still ongoing.

## 5. Insulin Resistance and Endothelial Dysfunction

As mentioned above, NAFLD may be considered the liver expression of metabolic syndrome and IR is the mayor pathophysiological implicated in the development of this syndrome [15]. The nitric oxide (NO) production is strictly influenced by the insulin activity, mediating not only the vasodilatation but also the anti-inflammatory, antithrombotic, and antifibrogenic properties of endothelium. The worsening of IR is related to the severity of endothelial dysfunction (ED), whose implication has been evaluated in different models of liver disease [96]. Following the loss of its capacities, ED contributes and promotes the structural and functional changes of liver circulation, impairing the regeneration after liver injury and contributing to progression of liver disease. NO is constitutively synthesized by endothelial nitric oxide synthase (eNOS), whose production of NO plays a fundamental role in the systemic vascular tone, regulating the blood pressure and renal control of extracellular fluid [97]. Insulin signaling leads indirectly to the activation of eNOS through the phosphokinase pathway PI3K/Akt, which may be impaired in the presence of an IR condition, while other pathways remain unaffected, as the Ras/MAPK pathway involved in the control of cell proliferation [98]. The result of this metabolic imbalance reflects the characteristics of ED including the decrease of eNOS activation and then the NO production.

The NO production is also driven through the inducible isoform of NOS (iNOS) that in contrast to eNOS, after its activation, produces large amounts of NO until the exhaustion of substrates or cofactors. The iNOS expression may be upregulated under condition of stress via proinflammatory cytokines (TNF-*α*, IL1, and IFN-*γ*), whose expression are increased in obesity and insulin resistance [99]. The excessive NO formation by iNOS has been shown to be involved in several inflammatory and autoimmune diseases (septic shock, rheumatoid arthritis, osteoarthritis, and multiple sclerosis), but among these, it has been shown that iNOS activity can reduce insulin sensitivity via S-nytrosilation of insulin receptor and modulation of PPAR-*γ* activation [100, 101]. Furthermore, the overexpression of iNOS contributes to ED with the loss of its anti-inflammatory function. In fact, an impaired generation of NO may promote the production on superoxide and hydroxyl radical intermediates, constituting a state of oxidative stress, which results in cellular apoptosis [102]. In this way, the ED may promote or worsen an inflammatory state, also known as “low-grade inflammation,” that leads to the progression of NAFLD liver injury [103]. Recently, in a mouse model fed with a high-fat diet for 6 weeks, the reduction of the eNOS activity was demonstrated in the early stages of NAFLD, with the result of reducing NO bioavailability, together with an increased oxidative stress and cyclooxygenase activity, resulting in an increase of hepatic vascular resistance unrelated to inflammation or fibrosis [104]. The same evaluation was assessed after cyclooxygenase inhibition therapy with thromboxane receptor antagonist, finding an improvement of endothelial dysfunction. These findings support the fact that the endothelial dysfunction takes part in the pathophysiological process of NAFLD from the early stages and oxidative stress contributes to vascular dysfunction, paving the way for new promising therapeutic strategies. The main pathophysiological mechanisms described above are summarized in Table 1.

## 6. Clinical Studies in Humans

Although several clinical evidences have highlighted the pathogenic correlation between oxidative stress and obesity-associated metabolic syndrome, its components (hypertension, obesity, and dyslipidemia), and/or diabetes [105–109], which are strictly associated with the presence of NAFLD, fewer clinical studies have investigated the association of oxidative stress and its extent with NAFLD itself. Some of these studies have examined the systemic oxidative alterations by directly evaluating various direct biomarkers of oxidative stress, whereas other studies have investigated the “clinical effects” of such alteration on the cardiovascular system of the patients with steatosis (i.e., with the evaluation of intima-media thickness, epicardial fat measurement, etc.). Others have correlated both serum biomarkers and clinical signs with steatosis and its extent. Moreover, of particular interest are the studies on gut-liver interactions and endothelial dysfunction, oxidative stress, and nitric oxide (NO) metabolism impairment, in particular throughout endothelial nitric oxide synthase (eNOS) activity derangement.

### 6.1. Studies Evaluating Oxidative Stress Markers

A study by Palmieri et al. demonstrated that serum vitamin C and alpha-tocopherol concentrations were lower and lipid peroxides (thiobarbituric acid reactive substances (TBARS)) were higher in patients with steatosis and metabolic syndrome in contrast with controls. The study concluded that patients with metabolic syndrome and steatosis exhibited a decreased antioxidant protection and increased lipid peroxidation [110]. On the same line, a recent paper by Stiuso et al. demonstrated that, not only were there two types of patients, one with higher basal severity of liver steatosis inflammation and fibrosis and the other with milder histological presentations, differing in the extent of oxidative stress (measured with TBARS, nitrite, superoxide dismutase, and catalase), but also that these evaluations could be useful as a prognostic marker of response to an antioxidant treatment [111]. As is clearly explained by the abovementioned pathophysiological mechanism of NAFLD itself (the classical “two-hit” theory [16] or the more up-to date “multiple parallel-hit” theory [17]), the oxidative stress represents the core mechanism at the basis of the “second hit” which conducts to the formation of reactive oxygen species (ROS). This mechanism is a consequence of the excess of fatty acids in the hepatic cells, their energy depletion and consequent mitochondrial dysfunction leading to increase in oxidative stress and, finally, cellular damage [106]. These mechanisms have been demonstrated by basic science studies on animal experimental models but have also found confirmations in humans [112, 113], giving a precise insight to the importance of the oxidative stress in the pathophysiology of NAFLD. Moreover, these theories found a “clinical” confirmation from the studies that evidenced the association between metabolic syndrome and also its components (hypertension, obesity, and dyslipidemia) taken individually [114–116].

Finally, even more interest in the last few years has risen on two other mechanisms of liver damage in which a major pathogenic role is played by oxidative stress: the gut-liver axis and the endothelial dysfunction. As far as gut-liver axis, recently, some studies have confirmed the already cited findings of experimental animal models in which intestinal dysbiosis is a factor leading to an increase of lipotoxicity. This occurs, as in animal models, via the stimulation of Toll-like receptors (TLRs) and the consequent activation of inflammasomes that induce cellular injury also by increasing oxidative stress. In particular, it has been demonstrated that in patients with NAFLD, the gut-microbiota impairment (increase in Bacteroidetes, decrease in Prevotella spp.) is associated with worse presentations of NASH [117]. Moreover, abundance in ethanol-producing bacteria was also observed in patients with NAFLD, indicating that this issue may lead to an oxidative stress increase and liver injury by “endogenous” production of ethanol in patients with NASH [93].

Finally, also in the frame of endothelial dysfunction, in which, as discussed above, the imbalance in eNOS and iNOS functionality leads to increase in oxidative damage of the liver vasculature leading to inflammation and fibrosis, our group recently published a paper demonstrating that systemic eNOS impairment is significantly associated with NAFLD [118].

## 7. Conclusions

The present review was aimed at describing the results of the most important studies focused on the explanation of possible pathogenetic theories of oxidative stress as the most important acting factor in all the biological hits able to determine the development and worsening of the complex clinical picture of NAFLD. For this reason, at this level of knowledge, the most important problem is the interpretation and correlation of some results obtained through the clinical studies. A lot of these are difficult to translate in routine clinical practice because of a loss of strong evidences that support their application in the therapy of this disease. Certainly, we must admit that the evolution in the comprehension of the mechanisms that support NAFLD has been very fast in the last decade, and at the same time, the analyzed fields represent some of the most promising topics of scientific research of the future.

## Figures and Tables

**Table 1:** Overview of the major pathophysiological mechanisms involved in oxidative stress in NAFLD addressed within the present paper, with the corresponding references.

Pathophysiological mechanisms of oxidative stress	Mechanism (with references)	
	Increasing prooxidant	Decreasing antioxidant
Mitochondrial dysfunction	Impairment of oxidative capacity of ETC, resulting in an “electron leakage” [21]	GSH depletion with reduction of GPx activity [41]
	Accumulation of Cer and DAGs due to incomplete betaoxidation of acyl-carnitine [34]	Reduced activity of MnSOD, polymorphism C47T of SOD2 gene [42]
	mtDNA mutation [25]	Impaired activity of cytochrome C [35]
	Production of reactive aldehydes (MDA, 4-HNE) through lipid peroxidation [43]	
	Increase activity of CYP2E1, polymorphism of C2 allele [37–40]	

ER stress	Prolonged activation of UPR leading to (i) overexpression of ERO1 [49] (ii) calcium disruption due to reduced activity or SERCA [55] (iii) upregulation of CHOP mediated by activation of PERK and ATF6 [50]	Prolonged activation of UPR leading to (i) inhibition of Nrf2 by PERK resulting in the depletion of GSH [59]

Iron metabolism derangements	Disruption of peroxisomal membrane [66]	GSH depletion—decreased GPx efficiency [67]
	Enhanced iNOS expression via NF-κB activation [67]	Inhibition of HO-1 by activation of BACH-1 [70–74]
	Production of reactive aldehydes (MDA) through lipid peroxidation [64]	Iron actin as a direct competitive antagonist of antioxidant enzymes [69]

Inappropriate inflammatory response mediated by GUT-liver axis	Upregulation of proinflammatory pathways and NADPH oxidase system due to bacterial and bacterial product translocation [80, 81]	Lack of inhibition of inflammatory response by NPRL-3 and -6 [85, 86]
	Activation of inflammasomes resulting in cleavage of cytokines precursors (pro-IL1*β*, pro-IL18) [82]	
	Endogenous alcoholic production by alcohol-producing bacteria [89]	

Insulin resistance and endothelial dysfunction	Upregulation of Ras/MAPK pathway involved in cell proliferation [94]	Decrease of eNOS activation due to IR [94]
	Enhanced iNOS activity due to increase expression of proinflammatory cytokines in IR [96]	

4-HNE: 4-hydroxy-2-nonenal; ATF6: activating transcription factor 6; BACH-1: BTB and CNC homology 1; Cer: ceramides; CHOP: C/EBP homologous protein; CYP2E1: cytochrome P 450 2E1; DAGs: diacylglycerols; ER: endoplasmatic reticulum; ERO1: ER oxidoreductin 1; ETC: electron transport chain; GPx: glutathione peroxidase; GSH: glutathione; HO-1: hemoxygenase 1; IR: insulin resistance; MDA: malondialdehyde; MnSOD: manganese superoxide dismutase; NPRL: NOD-like receptor protein; Nrf2: nuclear factor- (erythroid-derived 2-) like 2; PERK: protein kinase RNA-like ER kinase; SERCA: sarco/endoplasmic reticulum CA^2+^-ATPase; UPR: unfolded protein response.
