# Corrigendum to “Role of Oxidative Stress in Pathophysiology of Nonalcoholic Fatty Liver Disease”

**DOI:** 10.1155/2021/9757921

**Published:** 2021-07-28

**Authors:** Mario Masarone, Valerio Rosato, Marcello Dallio, Antonietta Gerarda Gravina, Andrea Aglitti, Carmelina Loguercio, Alessandro Federico, Marcello Persico

**Affiliations:** ^1^Internal Medicine and Hepatology Division, Department of Medicine, University of Medicine of Salerno, Salerno, Italy; ^2^Hepatogastroenterology Division, University of Campania “Luigi Vanvitelli”, Naples, Italy

In the article titled “Role of Oxidative Stress in Pathophysiology of Nonalcoholic Fatty Liver Disease” [1], there was an error in the legend of Figure 1, which should be corrected as follows:

“C oxidase (VI complex)” should be corrected to “C oxidase (IV complex).”

The authors apologize for the error.
